# The alcohol dehydrogenase isoenzyme (ADH I) as a marker of intrahepatic cholestasis of pregnancy

**DOI:** 10.1038/s41598-022-15532-9

**Published:** 2022-06-30

**Authors:** Joanna Piechota, Wojciech Jelski, Karolina Orywal, Barbara Mroczko

**Affiliations:** 1 2nd Department of Obstetrics and Gynecology, Medical University of Warsaw, Warsaw, Poland; 2 Department of Biochemical Diagnostics, Medical University, Waszyngtona 15 A, 15-269 Bialystok, Poland; 3 Department of Neurodegeneration Diagnostics, Medical University of Bialystok, Bialystok, Poland

**Keywords:** Biomarkers, Enzymes

## Abstract

Intrahepatic cholestasis of pregnancy (ICP) is an important pregnancy liver disorder. The alterations of different enzymes activity in the hepatocytes in the course of liver diseases are reflected in an increase in the activity of the corresponding enzymes in the blood. In present study we assayed the activity of alcohol dehydrogenase (ADH) and its isoenzyme in the serum of patients with ICP. Serum were collected from 100 pregnancies with ICP in the second or third trimester of pregnancy. Serum samples were also taken from 100 healthy pregnant women. The activity of ADH I was measured by spectrofluorometric method, ADH total was measured by photometric method. There was significant increase in the activity of ADH I (2.79 mU/l vs. 1.72 mU/l) and total ADH activity (1103 mU/l vs. 682 mU/l) in the sera of women with ICP compared to the healthy pregnant women. Importantly, the sensitivity and specificity of ADH I for diagnosis of ICP were 85% and 91%, respectively. Area under the Receiver Operating Curve for ADH I in ICP was 0.81. The activity of ADH I in the sera of women with ICP is statistically significantly increased, which may have a diagnostic significance for ICP patients.

## Introduction

Intrahepatic cholestasis of pregnancy (ICP) is a pregnancy-specific liver dysfunction disease, and its incidence is regional and shows familial aggregation and recurrence. The incidence of ICP varies from 5 to 15% in South America and 0.1% to 1.5% n other parts of the world^1^. The main symptom of ICP is pruritus, and it has been reported that 80% of patients develop itching at 30 to 32 weeks of gestation. The most common and useful biochemical test for diagnosing ICP is the total bile acid (TBA) level^2^. The greatest risk of ICP is associated with adverse perinatal effects, preterm delivery and an increased risk of sudden fetal death in the uterus^3^. Therefore, improving the prognosis of ICP depends primarily on early diagnosis and appropriate treatment by detecting appropriate markers in high-risk women to facilitate rapid interventions. Current diagnostic methods such as total bile acid etc. suffer from several disadvantages and cannot accurately predict the clinical stage of ICP.

Some studies have found that changes in enzyme activity in liver cells in the course of hepatic diseases are reflected by the alterations of its activities in the sera^4,5^. For example alcohol dehydrogenase (ADH) and its isoenzymes and aldehyde dehydrogenase (ALDH) in the sera are an indicators of liver cell injury^6^. These enzymes, apart from the oxidation of ethanol, play an important role in the metabolism of various key biological compounds (e.g. retinol). In our previous study, we have found that the total ADH activity was elevated in the serum of women with ICP. The increase in total ADH activity correlated with ADH I may be caused by the release of this isoenzyme from damaged liver cells in pregnant women. These preliminary studies of the activity of ADH isoenzymes allowed us to find a statistically significant increase in the activity of ADH class I and ADH total in the serum of women with ICP^7^.

The current research is a continuation of our previous study, we defined diagnostic criteria which include diagnostic sensitivity, specificity, positive predictive value (PPV) and negative predictive value (NPV) and the receiver operational characteristic curve (ROC) of the tested enzymes. These results may prove useful in the evaluation of alcohol dehydrogenase and aldehyde dehydrogenase as candidate ICP markers in pregnant women.

## Materials and methods

### Material

The Human Care Commission of the Medical University of Bialystok has approved the current research (Approval No. R-I-002/434/2017). Researchers obtained informed consent to study all patients.

Serum samples from routine biochemical tests from 100 pregnancies were used in the study (range age 18–39 years) complicated by ICP in the second or third trimester of pregnancy hospitalized in the 2nd Department of Obstetrics and Gynecology, Medical University of Warsaw (Poland). 42 women were pregnant for the first time, 39 for the second time and 19 for the third time. None of the women had ICP in previous pregnancies. Diagnosis was performed on the basis of clinical and laboratory investigations (total bile acid concentration, transaminases activities. Inclusion criteria were: skin pruritus beginning in the second/third trimester of pregnancy and an increase concentration of serum total bile acids (10–39 µmol per l). Exclusion criteria were: hepatic viral infection (HAV, HBV or HCV) and chronic liver diseases, hemolysis elevated liver enzymes low platelets, acute fatty liver of pregnancy, pre-eclampsia. None of the pregnancies were receiving rifampicin at least 1 year before serum collection. Blood samples from pregnant women were taken before treatment and possible surgery.

Tested group were compared with 100 healthy pregnant women (range age 18–41 years) in the second or third trimester of pregnancy. 40 women were pregnant for the first time, 43 for the second time and 17 for the third time. None of the women had ICP in previous pregnancies. The healthy controls were recruited from the same geographical location and ethnics populations as the patients and were not from the hospital. Control subjects were volunteers and were defined as those with normal results of all physical, blood examinations. None of the women consumed any alcohol. The analysis of the general clinical data of both groups is presented in Table 1.

**Table 1:** The analysis of the general clinical data of both groups.

	Pregnancies with ICP (n = 100)	Healthy pregnant (n = 100)
Gestational weeks	16–36	15–36
Concentration of TBA (mean)	33.4 µmol/l	4.2 µmol/l
Activity of alanine aminotransferase (mean)	106 U/l	51 U/l
Activity of aspartate aminotransferase (mean)	82 U/l	44 U/l
Activity of γ-glutamyltransferase (mean)	40 U/l	37 U/l
Pruritus	++/+++	–

### Methods

All methods were performed in accordance with the relevant guidelines and regulations as well as the recommendations of the authors of the methods.

#### Determination of the activity of class I ADH isoenzymes

The determinations were performed on a Shimadzu RF-6000 spectrofluorophotometer (Shimadzu Germany) at excitation wave 316 nm and emission wave 370 nm. Class I ADH isoenzyme activity was measured using fluorogenic substrate (4-methoxy-1-naphthaldehyde) in the reduction reaction described to Wierzchowski et al*.*^8^. The assays were performed in a reaction mixture containing sodium phosphate buffer (2.69 ml of 0.1 M pH 7.6), NADH (100 µl of 1 mM), substrate (150 µl of 300 µM), and serum (60 µl).

#### Determination of total ADH activity

The reduction of substrate was monitored at 440 nm on a Shimadzu UV 1202 spectrophotometer (Shimadzu, Germany). Total alcohol dehydrogenase activity was measured by the photometric assay with substrate (p-nitrosodimethylaniline)^9^. The reaction mixture (2 ml) contained 1.8 ml of a 26 µM solution of substrate in 0.1 M of sodium phosphate buffer, (pH 8.5) and 0.1 ml of a mixture containing 5 mM NAD and 0.25 M n-butanol and 0.1 ml of serum.

#### Determination of diagnostic values

The diagnostic specificity and sensitivity, the predictive value, the negative value, and the ROC curve, were calculated using GraphRoc for Windows (University of Turku, Finland) and the following equations^10^.

#### Statistical analysis

Statistical analyses were carried out using the STATISTICA12.1.PL program (Statsoft Poland). Initial statistical analysis (Chi-square test) showed that the distribution of ADH and ALDH activity was not consistent with the normal distribution. Therefore, the Mann–Whitney test for non parametric data was used for comparison between groups. Statistical significant differences were defined as *p* < 0.05.

## Results

The activities of total alcohol dehydrogenase, and isoenzymes class I of ADH in the serum are demonstrated in Table 2.

**Table 2:** Serum activity of ADH in ICP women and healthy pregnant.

Tested group	ADH I Median Range Mean	ADH total Median Range Mean
Pregnancies with ICP (n = 100)	2.79	1103
	0.76–5.46	354–2605
	2.58	1072
Healthy pregnant women (n = 100)	1.72	682
	0.51–4.18	269–1526
	1.51	648
	*p* < 0.05*	*p* < 0.05*

Data are expressed as mU/l.

*p*, pregnancies with ICP versus healthy pregnant women.

*Statistically significant differences between suitable groups.

The serum level ADH I in ICP patients was 2.79 mU/l. While the serum level of ADH I in the normal control group was 1.72 mU/l. Compared with the control group, ADH I were remarkablely (about 62%) raised (*p* < 0.01). The total alcohol dehydrogenase activity was significantly higher (38%) in pregnancy women with ICP than in the healthy pregnant women (*p* < 0.01). The median total activity of ADH was 1103 mU/l in women with ICP and 682 mU/l in control group. These results are comparable to those of our previous research.

Table 2 shows the diagnostic criteria for ADH total and ADH I. It was shown that the sensitivity and specificity of ADH I was higher than the values of total ADH. The cut-off value, sensitivity and specificity of ADH I in serum were 3.82 mU/l, 85%, and 91%, respectively. It was found that both the positive predictive values and the negative predictive values were also the highest for ADH I. PPV, NPV for ADH I were 88% and 82% respectively. More details are shown in Table 3.

**Table 3:** Diagnostic criteria for ADH total and ADH I for ICP.

Tested enzymes	Cut-off (mU/l)	Diagnostic sensitivity (%)	Diagnostic specificity (%)	Positive predictive value (%)	Negative predictive value (%)
ADH total	1456	74	80	81	77
ADH I	3.82	85	91	88	82

The cut-off points were obtained from a study of a health population (95th percentile).

**Figure 1:**
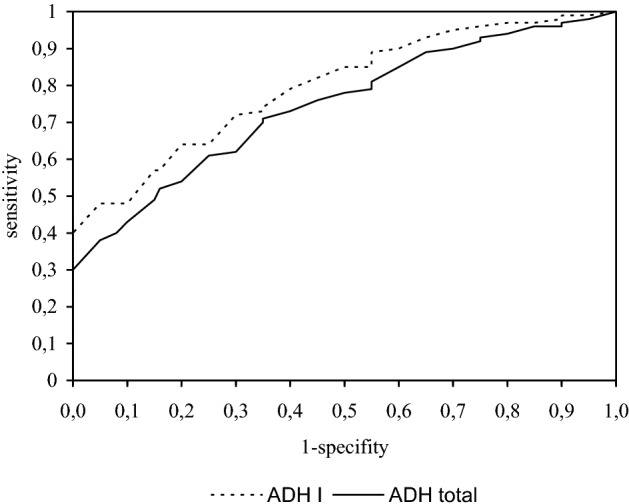
Areas under ROC curves for ADH I and ADH Total. ADH I, Area = 0.8103, SE = 0.0639.ADH total, Area = 0.7735, SE = 0.0682.

The ROC curve illustrates the relationship between diagnostic sensitivity and specificity (see Fig. 1). The area under the ROC curve for ADH I (0.81) was found to be higher than the ROC area of total ADH (0.77).

## Discussion

Intrahepatic cholestasis of pregnancy is an important pregnancy liver disorder. At present, the incidence of ICP is considered to be linked to genetic factors, hormonal factors and environmental factors, but its etiology and pathogenesis remain largely unclear^11^. Early diagnosis and treatment is of paramount importance given the serious adverse effects on the fetus. The patients should seek early intervention to avoid the adverse effects of pregnancy such as premature delivery, perinatal diseases and even death. In recent years, no there has no progress in the development of novel diagnostic options of ICP. The diagnosis of ICP is based on the symptoms of itching and elevated levels of bile acids. Liver biopsy is rarely performed to confirm diagnosis. However when done, it usually shows cholestasis with preserved portal channels. Total serum bile acids are the most commonly used biomarkers for ICP in medical practice. Certain components of the serum bile acid profile can provide more detailed information than TBA in diagnosing disease, determining its severity, and monitoring response to treatment. However so far, the diagnostic accuracy of TBA in intrahepatic cholestasis in pregnancy could not be accurately estimated. There have been insufficient studies to allow an accurate estimation of the accuracy of the components of the serum bile acid profile^12^. Liver function tests are commonly performed when ICP is suspected, but their normal upper limits in pregnant patients are still discussed. Liver biochemistry such as aminotransferases (alanine and aspartate) are also often elevated, and various other causes of liver dysfunction must be ruled out^13^. Maternal prognosis is benign in ICP and symptoms, and abnormal hepatic biochemical tests resolve quickly after delivery. Some patients have elevated liver function tests and should be further investigated for any other cause. In one large cohort study, patients with intrahepatic cholestasis during pregnancy showed a higher incidence of hepatobiliary disorders later in life and, hepatitis C, hepatic fibrosis, chronic hepatitis or cirrhosis, and cholangitis or gallstones observed more frequently in these women compared to the overall population^14^. Hence it is necessary to find markers that can detect intrahepatic cholestasis of pregnancy as early as possible. In the serum, we observe a reflection of the changes in enzymatic activity in the liver cells altered by ICP.

Human liver cells are rich in various isoenzymes of alcohol dehydrogense. ADH is a family of enzymes that has been divided into several classes. The first two classes in humans are found mainly in the liver cells. Class I ADH is a classic hepatic alcohol dehydrogenase, but is also found in minor amounts in the gastrointestinal tract, kidneys and lungs^15^. The ADH profile of ICP patients has not been previously studied by other investigators. In our previous study we have shown that the serum total alcohol dehydrogenase activity has been changed in the course of ICP. The increase of total ADH was positively correlated with ADH I so the cause for the increase of serum total alcohol dehydrogenase activity in the course of ICP is an elevation of class I ADH isoenzyme^7^. Serum ADH levels are increased in intrahepatic cholestasis of pregnant and our aim was to investigate alcohol dehydrogenase isoenzymes in pregnant women with ICP as makers of this disease increasing the number of tested women and setting diagnostic indicators. One hundred women with intrahepatic cholestasis of pregnancy and 100 healthy pregnant women were included in this cross-sectional study. In the group of women with ICP, the concentration of TBA was 10–39 µmol per l, the levels of alanine and aspartate aminotransferase were high, with a more apparent elevation of alanine than aspartate aminotransferase (mean activity 104 and 75 U/l respectively). In the control group the concentration of TBA was < 10 µmol per l. The mean activities of alanine and aspartate aminotransferases were 47 and 41 U/l, respectively. Pruritus was observed in women with ICP and no pruritus was observed in healthy pregnant women.

An excellent marker should have a high specificity, i.e. it must not be detectable in healthy people and be highly sensitive, i.e. very small amounts can be detected at an early stage. None of the known diagnostic markers have met the criteria of 100% sensitivity and specificity yet. In our current paper we have shown that during the course of intrahepatic cholestasis of pregnancy diagnostic sensitivity was highest for ADH I (85%) and ADH total (74%).

The probability with which disease exists in the case of a positive test result is the predictive value for positive result. While the probability of no disease in the case of negative test results is the predictive value for negative results. In our studies, we showed that ADH I has a high positive predictive value (88%) and a negative predictive value (82%). The ROC curve, which is a plot of sensitivity/specificity, is the most important criterion for disease markers. The clinical usefulness of the tested substances is indicated by the area under the ROC curve. The better the disease marker (more sensitive and specific), the greater the area under the ROC curve. In present study, we found that ADH I has a larger ROC curve area than total alcohol dehydrogenase.

Currently, the discussion on the diagnosis, prognosis and therapeutic management of intrahepatic cholestasis in pregnancy is still open, and doctors need new information about this disease. For the first time, all diagnostic criteria for alcohol dehydrogenase and aldehyde dehydrogenase have been tested in women with intrahepatic cholestasis of pregnancy. The obtained results suggest a potential diagnostic role of ADH (especially ADH I) as ICP markers, but further research and confirmation by a prospective study are are needed.

## Data Availability

The datasets used and/or analyzed during the current study available from the corresponding author on reasonable request.
